# Some Observations Relative to the Angustura Bark

**Published:** 1797

**Authors:** Thomas Masterman Winterbottom

**Affiliations:** Physician to the Settlement at Sierra Leone.


IV. *Some Obſervations relative to the Anguſtura Bark. Communicated in a Letter to Dr.* Simmons, *by* Thomas Maſterman Winterbottom, *M. D. Phyſician to the Settlement at Sierra Leone.*

IN compliance with your requeſt I ſend you a ſhort account of my experience relative to the Anguſtura bark, with a few of the caſes in which I have employed it. A long time is, in general, required before the merits of a new medicine can be duly aſcertained; for while ſome extol it to the ſkies as a cure for every diſeaſe, others are equally fond of depreciating the powers which it actually poſſeſſes. Experiments frequently repeated are therefore neceſſary, before we can form a juſt eſtimate of the effects of any remedy. In the preſent inſtance I ſhall ſcarcely be accuſed of precipitancy, or of giving an opinion drawn only from a few imperfect caſes. During my ſtay in Sierra Leone I have uſed upwards of fifty pounds weight of the Anguſtura bark in powder; and from every trial hitherto made of it, I think myſelf warranted ranted to recommend it as an active and valuable medicine.

In ſeveral comparative trials made with the Anguſtura and common Peruvian barks, in regard to their febrifuge and tonic powers, I have always found the former to be equally efficacious with the latter, and that frequently in ſmaller doſes. In thoſe caſes, however, where it is neceſſary to give this medicine in ſubſtance, and in large doſes, as in the remittent fever, with a view to put a ſtop to the return of the paroxyſm, the Anguſtura bark could not always be given for a ſufficient time, without exciting nauſea; but where this effect was not produced, I have truſted the courſe of a remittent fever to the Anguſtura with the ſame confidence as to the Peruvian bark, which laſt is uſually conſidered as a ſpecific for that diſeaſe. It muſt, however, be obſerved, that in the caſes of fever where the Anguſtura bark was employed, the doſes were perhaps larger than might be abſolutely neceſſary; but the fever of this country is uſually ſo rapid in its progreſs, that if the paroxyſms be not ſoon put a ſtop to, the remiſſions become obſcure, or ſcarcely perceptible, and the patient is ſuddenly carried off. I did not venture, therefore, to uſe it in ſmaller doſes than what I had from experience found neceſſary to be given of the Peruvian bark; nor did I conſider my patient as ſecure unleſs he had taken, during the time of a remiſſion, as much of it as his ſtomach would bear.

Towards the decline of a fever, when debility is the chief ſymptom, I prefer the uſe of the Anguſtura bark, in infuſion, to a farther continuance of the Peruvian bark; this change is generally very agreeable to the patient; the infuſion ſits eaſy on the ſtomach, and is attended with the moſt beneficial effects in reſtoring the ſtrength and appetite. I have alſo found Anguſtura bark very effectual in the cure of intermittents: but as theſe moſt commonly occur among the ſeamen and Nova Scotian ſettlers, who are not eaſily induced to take a diſagreeable medicine for any length of time, I have been almoſt always obliged to ſubſtitute the arſenical ſolution in place of the bark.

### CASE I.

July 15, 1792. Mr. A. aged 38, of a ſallow complexion, with light brown hair, was, on on the evening of the 13th, attacked with a remittent fever, the ſymptoms of which were very ſlight, and went off towards morning, but returned at night with greater violence; he then took a doſe of James's powder, which vomited and purged him ſeveral times during the night. At preſent, (four, p. m.) he complains of pain in the head, eſpecially over the eyes, and great depreſſion of ſtrength; his pulſe is 100, and quick; his ſkin clammy, and rather hot; he is affected with nauſea, and inclination to vomit, on ſitting up or attempting to walk: tongue foul, much thirſt. He was ordered to take at bed-time a draught, with Tinct Opii, gutt. xxx. Sp. Nitr. d. gutt. xl. Aq. font. ℥ſs.; and Pulv. Cort. Anguſt. ʒſs. every hour, beginning early in the morning.

16. (Ten, a. m.) He has paſſed rather an uneaſy night, and ſeems very irritable, being eaſily affected by the ſlighteſt noiſe, &c. Skin rather cooler; pulſe 100; other ſymptoms as before. The powder and draught to be repeated.

17. Had an exacerbation of fever yeſterday at two, p. m.; pulſe 120, and quick; ſkin very hot and dry; pain of the head much increaſed; great reſtleſſneſs and anxiety. Took yesterday six drachms of Angustura bark in powder; the medicines to be repeated.

18. Ten, a. m. Has passed a very restless night; had a return of fever yesterday, at the same time as the day before, with nearly the same symptoms, but which went off towards evening, with a clammy, fœtid perspiration. He is rather costive; pulse 96; much nausea was produced by the medicine, of which he took yesterday an ounce, and retained it, though with great difficulty. Twenty grains of *Kali pp.* were directed to be added to each dose of the powder, and a large spoonful of lime-juice to be taken immediately after it.

Six, p. m. The paroxysm returned much later this afternoon than usual, and was very slight. At present, his skin is cool and moist; his thirst less; head-ach abating; pulse 100, and pretty soft. He vomited after one dose of the powder, but has retained near an ounce of it to-day; feels much less nausea since he has used the effervescing draught. Powders and anodyne draught to be repeated; to take also three aloetic pills at bed-time.

19. Has passed a tranquil night, but without sleep; feels much easier this morning; a slight pain still remains over his eyes; his tongue is is moiſt, but he has a diſagreeable taſte in the mouth; his ſkin is cool and moiſt: he had one ſtool from the pills. The medicine being taken with great difficulty, it was ordered to be omitted after this day. In place of it he was deſired to take half a drachm of Peruvian bark, in powder, every four hours, beginning in the morning. The draught was repeated.

24. He has had no return of fever ſince the 18th, and has no complaint at preſent but of languor and want of ſleep, to which he is often ſubject when in health. His appetite returns.

### CASE II.

Mr. N. aged 40, of a dark complexion, and of a robuſt habit of body, was taken ill of a fever at the ſame time as the laſt patient. The chief ſymptoms in Mr. N.'s caſe were great proſtration of ſtrength, with reſtleſſneſs and anxiety. His pulſe, which in health beat only ſixty-four times in a minute, and was remarkably full and ſtrong, during the paroxyſm never exceeded eighty-ſix in a minute. He took every day, for a week, ten drachms of the powder of Anguſtura bark, without experiencing the the least nausea. The Angustura bark appeared to stop the recurrence of the paroxysms sooner in this case than the former; and the patient's strength returned much more speedily.

From the beginning of his illness till some time after the perfect recovery of his health, his urine was of a very dark colour, like the grounds of coffee.

### CASE III.

March 9, 1794. J. Fargrain, sailor, aged 30, of rather a sallow complexion, has felt, for two days past, much languor and lassitude, with pains of the head and back, and frequent slight chills, which usually came on towards evening, and abated in the morning. At present he has an acute pain over his forehead, and a dull pain or heaviness across his loins; much giddiness on standing in an erect posture; tongue rather white; a bad taste in the mouth; much thirst, and a considerable depression of strength. He was directed to take in the evening, *Antimon. Tartarisat.* gr. ij. and *Pulv. Ipecac.* ℈j.; and at bed-time, *Tinct. Opii.* and *Vin. Antimon.* āā. ʒss.

10th. (Ten,

10th. (Ten, a. m.) The vomit operated, and evacuated much foul bilious matter. He paſſed rather a reſtleſs night, but feels conſiderably relieved this morning; pulſe 86; ſkin moiſt and pretty cool; a ſlight pain ſtill remains over his eyes; ſome thirſt; pain of the back almoſt gone. He was directed to take half a drachm of powder of Anguſtura bark every hour, and at night repeated the tincture of opium and antimonial wine.

11th. (Ten, a. m.) Had an exacerbation of fever laſt night, attended with ſevere pain of the head, and felt much anxiety and reſtleſſneſs during the night. At preſent, pulſe 100, and ſoft; ſkin hot, but moiſt; much thirſt; he had one ſtool this morning. He took yeſterday ſix drachms of the Anguſtura bark, but vomited after the laſt doſe; he was directed to take a drachm of the powder every hour. The tincture of opium and antimonial wine were repeated at bed-time.

12th. He has reſted pretty well all night, and felt no return of fever; had four ſtools yeſterday, and two in the night. Pulſe 80, ſoft, and rather full; ſkin cool; tongue pretty clean, but dry; thirſt abated; a ſlight head-ach ſtill remains; urine rather high coloured and and tranſparent. He took yeſterday an ounce and a half of Anguſtura bark; the other medicines were repeated at bed-time.

13th. He has paſſed a reſtleſs night, attended with great anxiety, but has had no return of fever. At preſent, pulſe 88, ſoft, and pretty full; ſkin cool; has ſtill a ſlight pain over his eyes; he has ſome return of appetite, and is able to ſit up a little in bed; his urine is ſtill high coloured, and without cloud or ſediment. He took yeſterday an ounce and a half of Anguſtura bark; the other medicines were repeated.

20th. He has continued the uſe of the Anguſtura bark ſince the 13th, but in doſes gradually diminiſhed. He has no complaint at preſent but from weakneſs. His urine is high coloured as before. He was deſired to take half a drachm of Anguſtura bark three times a day, for a few days longer.

### CASE IV.

Mr. P. aged 45, of a fair complexion, with light brown hair, was ſeized, on the 23d of March, 1794, at five, p.m. with ſevere pain over his eyes, giddineſs, nauſea, and retching. He took immediately *Antimon. Tartariſat.* gr. ij. and *Pulv. Ipecac.* ℈j; and, at bed-time, thirty drops of tincture of opium.

24th. (Ten, a. m.) The emetic operated ſeveral times; he paſſed the night without ſleep; feels very languid at preſent, and complains of a ſevere pain of his head and back; his ſkin is hot and clammy; his pulſe 98, full and ſoft. He complains of great giddineſs and increaſe of head-ach on raiſing his head from the pillow. He was directed to take two ſcruples of the powder of Anguſtura bark every two hours; and at night to repeat the opiate

25th. He reſted very ill laſt night, which he attributed to the opiate: ſymptoms in other reſpects much the ſame as yeſterday. He complains of great thirſt; his tongue is very clammy, and covered with a white fur; ſkin hot but moiſt; pulſe 98, and pretty ſoft; body open. He took yeſterday ten drachms of Anguſtura bark, but vomited after the laſt doſe; the opiate to be omitted.

26th. He had ſome ſleep laſt night; the pain of his head is now leſs ſevere; the pain of his back is removed; he had three looſe ſtools during the night; pulſe 90, and ſoft; the the debility and giddinefs ftill very great. He took yefterday ten drachms of Anguftura bark, and had fome naufea from the laft dofe, but without vomiting.

27th. He flept pretty well laft night, and feels himfelf confiderably better to-day. His head-ach is almoft gone; pulfe 84, foft, and pretty full; fkin cool; tongue moift and clean. He has much giddinefs on attempting to fit up, and begins to naufeate the powder; he took, however, an ounce of it yefterday, and retained it: half a drachm of it was now directed to be given only every three hours.

30th. He complained only of debility, and his appetite was pretty good; he ftill continued, however, to take the Anguftura bark for fome days longer.

In dyfentery I have not had fo many opportunities of trying the effects of Anguftura bark, as in fever, for though dyfenteries fometimes occur here, they are not very frequent, nor, in general, obftinate. In thofe cafes, however, where the fymptoms ftill continued to harafs the patient, after the bowels had been fufficiently opened by faline and mercurial purgatives, the Anguſtura bark never failed to put a ſtop to the complaint. In theſe inſtances the powder was moſt commonly uſed, in doſes of from one to two ſcruples, and repeated three or four times a day, according to the circumſtances of the caſe. Sometimes an infuſion of the bark was given, prepared by macerating half an ounce or ſix drachms of it powdered, in a pint of water for twelve hours, in a gentle heat.

Diarrhœas occur frequently in this place, and are often of a very obſtinate nature, particularly when they attack thoſe who have been much debilitated by previous diſeaſe, or who, during their convaleſcence, have indulged in the uſe of improper food; or, as is more commonly the caſe, when they attack thoſe who cannot procure a diet ſufficiently nouriſhing. In all theſe caſes I have ſeldom or never found the Anguſtura bark to fail in putting a ſtop to the diſcharge. When the diarrhœa has continued for any length of time, and the ſtools have been frequent, ſmall, and attended with ſome degree of teneſmus, I have found it uſeful previouſly to check the diſeaſe, by exhibiting from fifteen grains to a ſcruple of *Pulv. Ipecac.* This was done, not in order to evacuate any acrid matter from the ſtomach, but with a view of exciting exciting an antiperiſtaltic motion of the ſtomach and inteſtines, or of counteracting their irre gular action, which had been probably kept up through habit. A ſimilar effect is frequently produced in long-continued vomitings, which, after reſiſting for a length of time the medicines uſually employed, are often ſpeedily removed, by briſkly exciting the proper motion of the inteſtines.

When the emetic has produced its full effect, the Anguſtura bark is to be given in doſes ſuited to the patient's age and ſituation; and ſuch is the effect of this mode of treatment, that in the courſe of a few days it commonly becomes neceſſary to adminiſter a gently opening medicine, to prevent coſtiveneſs from enſuing.

### CASE V.

J. Allen, aged 30, of a fair complexion; has been near a year in this country, during which period he has had two or three ſevere attacks of fever, which have reduced his ſtrength very conſiderably.

Sept. 22, 1793. He has been affected for four days paſt with ſevere and conſtant griping pains round the umbilicus, attended with frequent purging and tenefmus; his ftools are fmall, frothy, and chiefly compofed of blood and mucus; pulfe 100, quick, but rather foft; fkin tolerably moift. He labours under great debility, and is very anxious refpecting his recovery. He has taken, fince the firft attack, *Tinct. Opii* & *Vin. Antimon.* āā, gutt. xxx. and a pill, containing three grains of calomel. All thefe were repeated this evening.

23d. (Ten, a. m.) He has paffed a very reftlefs night; and has had feveral ftools, which were rather larger than ufual, and of a more natural colour; the tenefmus is abated; his pulfe 112, and quick; his tongue dry and brown.

He was directed to take half a drachm of Anguftura bark three times a day. The tincture of opium and antimonial wine were repeated at bed-time.

24th. He had fome reft during the night; and feveral ftools without tenefmus: the griping pains are now entirely gone; his fkin is cool; his tongue moift; his pulfe 80, and foft. The tincture of opium and antimonial wine were repeatd.

25th. He refted pretty well laft night; he has had only five ftools, and thefe were without griping or tenefmus. His weaknefs and anxiety are not much abated; pulfe 120, and quick, occafioned

ſioned by his ſitting up and walking a little about the room. The ſame medicines were repeated.

28th. He had only two ſtools to-day, which were perfectly natural. The pains of the bowels and other dyſenteric ſymptoms ſeem to be wholly removed.

October 2d. Debility is now the only remaining ſymptom of diſeaſe; he has, in general, two natural ſtools every day, and his appetite is returning. He was deſired to continue his medicines ſome time longer.

### CASE VI.

July 14, 1792. Capt. ———, has reſided a conſiderable length of time upon this coaſt, being chiefly employed in trading up ſmall rivers and creeks, by which his health has been greatly impaired. About two months ago he was ſeized with very ſevere dyſenteric ſymptoms, which have continued to haraſs him ever ſince; he has only been able to procure a few doſes of ſalts, but was ſomewhat relieved by them from the griping and teneſmus. At preſent he appears very much reduced; his countenance is ſallow; his ſtools are very frequent,

 ſmall,

ſmall, and frothy, and diſcharged with pain. He has frequent griping during the day, eſpecially after eating; pulſe feeble, and rather quicker than natural; ſkin pretty cool. He was directed to take in the morning Sal. Cathart. Amar. ℥i. in divided doſes, and after they ſhould have operated, to take half a drachm of Anguſtura bark, in powder, three or four times a day.

I did not ſee him again till ſome time afterwards, when he told me that he enjoyed better health than he had done ſince he came upon the coaſt. His complaint, he ſaid, left him after he had taken the powder three days, but he continued the uſe of it ſome time longer.

### CASE VII.

January 1, 1794, Mr. B. aged 40, of a ſallow complexion, with black hair, has been affected for ſix weeks paſt with ſevere griping pains, chiefly round the umbilicus, which are conſiderably increaſed after eating. He has alſo a conſtant teneſmus, and goes to ſtool not leſs than twenty times a day; the diſcharges are ſmall, mucous, and generally tinged with blood: his countenance is very unhealthy, pale, and and dejected; pulſe 90, and pretty ſoft; ſkin cool; much thirſt. He reſided a long time on the river Gambia, where he was affected with a remittent fever, which, after debilitating him conſiderably, changed to an intermittent; this latter was alſo very troubleſome for a long time, but quitted him before his arrival at Sierra Leone. The preſent complaint began with ſevere pain of the ſtomach, from which he was ſometimes relieved by opium and warm cordials; and the pain gradually deſcended lower, until it fixed round the umbilicus. Having been coſtive on the firſt attack, he took ſeveral purgatives. His conſtitution has ſuffered much injury by drinking ſpirits to exceſs. He was directed to take a ſcruple of Anguſtura bark, in powder, four times a day.

Jan. 3d. He feels himſelf relieved from the griping round the umbilicus ſince he has taken the Anguſtura bark; the teneſmus is alſo abated. The ſtools are leſs frequent, and chiefly mucous, without any mixture of blood: his appetite is improving, and he does not feel any return of pain after eating, as before. The uſe of the bark was continued.

6th. He had only two ſtools yeſterday, and one to-day; has now no complaint from his bowels, and his ftrength is returning. He was directed to take five grains of calomel in the morning, and to continue the ufe of the bark a few days longer.

It frequently happens, that perfons unaccuftomed to this climate, after fuffering a fevere fit of ficknefs, are apt to continue in a ftate of great weaknefs and irritability for a confiderable time, even for feveral months, without any vifible alteration. This moft ufually happens when the patient has refufed to take medicine during the fever, &c. or has not continued its ufe a fufficient time after the complaint has left him. The confequence is, that towards evening he generally becomes very reftlefs and uneafy; his fkin is dry, and hotter than ufual; his pulfe is not much affected while in a recumbent pofture, but on fitting up or ufing any motion, it is foon accelerated. Thefe fymptoms harafs the patient during the whole night, but are moftly carried off in the morning by a flight partial fweat upon the head and breaft. A flight chill fometimes ufhers in thefe fymptoms, and frequently there is pain in the forehead, or crown of the head.

The chief indication in ſuch caſes is to ſtrengthen the conſtitution, for which purpoſe I have employed a variety of tonic remedies, eſpecially Peruvian bark, Colombo, and Gentian; but I have never found any of theſe ſo ſpeedily efficacious as the Anguſtura bark in reſtoring ſtrength and appetite.

In a caſe of Hemicranium, which had continued ſome weeks in a very irritable habit, attended by a train of nervous ſymptoms, and where bliſters had procured only temporary relief, the diſeaſe was ſoon removed by an infuſion of Anguſtura bark, made by infuſing an ounce of the powder for two days in a pint of Madeira wine, of which two ounces were taken three times a day.

### CASE VIII.

Simon Elliott, a black man, aged 44, had been for a long time affected with a tertian intermittent. Before he applied to me the paroxyſms had ceaſed, but were ſucceeded by a large tumour on the left ſide, which projected conſiderably below the falſe ribs, and which was attended with a ſhort dry cough, dyſpnœa, and œdematous ſwellings of the lower extremities, mities, reaching nearly to the groin. The Squill pill, with calomel, produced no other effect than to render his mouth ſlightly ſore. The *Infuſ. Nicotian.* was then given to him, and proved a powerful diuretic, but though uſed very cautiouſly in ſmall doſes, it brought on head-ach, with dimneſs of ſight, and was therefore laid aſide. He was next ordered to take *Pulv. Cort. Anguſt.* & *Crem. Tart.* āā. ʒſs. three or four times a day, which kept his bowels moderately open, and increaſed the quantity of urine ſo conſiderably, that after continuing the uſe of the medicine for ten days, the ſwellings entirely diſappeared, and by perſiſting for ſome time longer in the uſe of the Anguſtura bark alone, he completely regained his ſtrength.

I have had but one opportunity of trying the Anguſtura bark in the form of enema; this was in the caſe of a gentleman who had been only a few months in the colony, and had ſuffered greatly from repeated attacks of fever. Having had a relapſe while in a ſtate of great debility, his ſtomach became ſo weak and irritable, as to reject, almoſt immediately, every thing he took. The efferveſcing draughts, joined with opium, produced only a temporary relief, and often failed even in that reſpect; his

ſkin

ſkin was hot and dry; his pulſe from 112 to 120, and ſmall; there was at the ſame time great reſtleſſneſs, and his voice was ſo weak, that he could ſpeak only in a whiſper. An enema, conſiſting of half an ounce of powder of Anguſtura bark, half a drachm of tincture of opium, and ſeven ounces of water, was directed for him twice a day; and at bed-time he took an effervefcing draught, with *Tinct. Opii*, gutt. xv. By theſe means the vomiting was checked almoſt immediately, ſo that he could retain a little nouriſhment; his pulſe beat ſtronger, and only 96 in a minute; his ſkin became moiſt, and a ſlight pain over his eyes, which he had before complained of, entirely went off; a ſmall degree of appetite alſo returned, and he appeared, upon the whole, to be recovering his ſtrength. Having a diſlike to the enema, it was omitted, after having been uſed near a week. After continuing nearly a week longer, with the ſame favourable ſymptoms, he ſuddenly fell into a ſtate of coma, which ſoon carried him off.

I have thus endeavoured to communicate to you whatever has occurred to me reſpecting the uſe of the Anguſtura bark; but ſeveral of my papers having been loſt or deſtroyed by the French,

French, when they paid us a visit at Sierra Leone, this account is thereby rendered more imperfect than it might have been. Neverthe-less, with all its defects, it may tend, in some degree, to prove that the Angustura bark is a valuable acquisition to the Materia Medica, and a medicine not unworthy of the praises which have been bestowed upon it.

*Free Town, Sierra Leone,*
*Dec.* 24, 1794.

